# Autobiographical memory coherence in emotional disorders: The role of rumination, cognitive avoidance, executive functioning, and meaning making

**DOI:** 10.1371/journal.pone.0231862

**Published:** 2020-04-20

**Authors:** Elien Vanderveren, Patricia Bijttebier, Dirk Hermans

**Affiliations:** 1 Center for the Psychology of Learning and Experimental Psychopathology, KU Leuven, Leuven, Belgium; 2 School Psychology and Development in Context, KU Leuven, Leuven, Belgium; University of St Andrews, UNITED KINGDOM

## Abstract

The ability to construct coherent narratives about significant personal experiences, commonly referred to as autobiographical memory coherence, has been related to various emotional disorders, though insight regarding mechanisms that might underlie this relation is scarce. The present study contributes to this growing body of research by examining the relation between memory coherence and both depression and PTSD and by investigating the role of rumination, cognitive avoidance, executive functioning, and meaning making in that relation in a large-scale community sample. The negative relation between memory coherence and both depression and PTSD could not be replicated, nor could the hypothesized negative relation between memory coherence and both rumination and cognitive avoidance be confirmed. In contrast, results indicated more memory coherence to be related to more rumination. Additional analyses in light of these surprising findings revealed that there was a significant indirect relation between memory coherence and both depression and PTSD-related symptoms through rumination. When the latter was controlled for, memory coherence was predictive of PTSD diagnosis and the hypothesized negative association with cognitive avoidance could be confirmed. In line with predictions, both executive functioning and meaning making were positively related to memory coherence. Theoretical and clinical implications are discussed.

## Introduction

With the number of people affected by emotional disorders worldwide exceeding 300 million [1], research that focuses on identifying predictors of such emotional problems is vital. A long tradition of research has focused on autobiographical memory, as it has been established to play a role in both the development and maintenance of emotional disorders, especially depression and post-traumatic stress disorder [2,3]. One characteristic of autobiographical memory in particular has received extensive attention over the course of the last three decades, namely memory specificity [4]. Besides their level of specificity, however, autobiographical memories differ along other features, such as their emotional intensity, the amount of detail they entail, or their coherence. A growing body of research has focused on the latter, commonly referred to as autobiographical memory coherence. The current study will contribute to this growing research interest by examining the relation between memory coherence and emotional disorders and by exploring mechanisms that could underlie this relation.

### Autobiographical memory coherence

Autobiographical memory coherence, also referred to as narrative coherence, can be situated within the broader literature of life narratives. In an attempt to process the multitude of experiences people encounter throughout their lives, they construct narratives [5,6]. Together, these narratives form a life story, which reflects a person’s unique personal history and forms the basis of their sense of self or identity [6,7]. The manner in which these narratives are integrated into an overarching life story is associated with various psychological outcomes. Being able to construct a coherent life story is, for instance, associated with feelings of continuity, purpose, and meaning in life [7,8]. In addition, it contributes to a healthy identity development [7–9]. Not only the coherence of the entire life story, but also the coherence of single events seems to be associated with psychological well-being. The latter is referred to as autobiographical memory coherence and will be the focus of the present study.

Autobiographical memory coherence refers to one’s ability to construct coherent narratives about personal experiences. Coherent narratives situate events in time and place and describe how the events unfolded in a chronological manner. In addition, they contain a clear theme that is elaborated upon, both factually and emotionally, and end in a resolution [10]. The ability to construct coherent narratives is related to psychological well-being, such as satisfaction with life, feelings of meaning and purpose, and experiencing positive relationships with others [11,12]. Additionally, memory coherence can act as a buffer against the impact of negative life experiences [13,14]. Vanderveren et al. [14] showed in a recent prospective study that students more adept at constructing coherent narratives reported to be less distressed following a future negative life experience compared to students less skilled in memory coherence.

In contrast, not being able to create coherent narratives has been related to symptoms of psychopathology. More specifically, various studies have shown low memory coherence to be related to depressive symptoms [15,16], PTSD [17, but 18], eating, and obsessive compulsive disorders [19]. Especially the coherence of narratives describing a negative experience seems to be associated with symptoms of psychopathology [16]. However, even though low memory coherence appears to be related to various psychological disorders, knowledge about mechanisms that might explain these relations is scarce. Nonetheless, such insight might have substantial clinical implications. Therefore, in a first attempt to increase our knowledge regarding such mechanisms, we will explore four potential correlates of memory coherence; meaning making, rumination, cognitive avoidance, and executive functioning.

### Meaning making

To identify potential correlates of memory coherence, we first delved into the broader literature on autobiographical narratives. Within this literature, the process of meaning making holds a central position. It refers to the universal process of making sense of life experiences. Especially in the case of negative life experiences, which are often discordant with one’s life goals and values, individuals are driven to create meaning in order to resolve the discrepancy between the current and desired situation [20]. One way through which people often attempt to process life experiences, is by creating narratives [5,6].

Being able to construct coherent narratives implies a certain degree of understanding of and insight into the experience; one has an understanding of how the events unfolded, feelings and thoughts evoked by the experience, how the experience has impacted their life etc. [10,21]. Constructing such coherent narratives is believed to promote meaning making [21,22], which has been shown to facilitate recovery and to be beneficial for both physical and psychological well-being [20,23–25]. So, driven by a need to understand and make sense of experiences encountered throughout our lives, people attempt to construct coherent narratives. Constructing such coherent narratives allows the individual to express and regulate emotions and thoughts related to the event and, eventually, come to some sort of resolution or closure. In contrast, not being able to construct a coherent narrative and, consequently, not being able to create meaning out of the experience, withholds the individual to move on and subsequently makes the individual vulnerable for psychological problems [21]. Taken together, the universal desire for meaning drives the creation of coherent narratives which in turn is predictive of successful meaning making.

### CaR-FA-X model

A second strategy to identify potential correlates of memory coherence, is by relying on the extensive knowledge gathered within a related research field, namely the domain of memory specificity. In 2007, Williams and colleagues [4] developed the CaR-FA-X model, which describes three important correlates of memory specificity; rumination, cognitive avoidance, and executive functioning. Given both the theoretical and empirical overlap between memory specificity and memory coherence [16,26], we propose that these constructs might be correlates of the latter as well. However, to understand how these constructs might relate to memory coherence, insight into the steps required to construct a coherent narrative is required.

The ability to create a coherent narrative about a particular personal event implies access to specific information regarding that experience (e.g., when and where did the event take place, sequence of events, thoughts and feelings at the time of the event, precursors and consequences associated with the event etc.). This specific information is generally assumed to be stored at the most concrete and specific level of the Self-Memory System (SMS), namely the level of event specific knowledge (ESK) [27]. Therefore, memory coherence, similar to memory specificity, can be situated at the level of ESK [26]. Gaining access to this most concrete layer in the SMS requires a vertical or top-down search process starting at the most abstract and general level (i.e., life time periods). Williams and colleagues’ CaR-FA-X model [4] describes three processes that can disrupt this search process: insufficient executive capacities, cognitive avoidance, and rumination. We hypothesize that, by disrupting this search process, the three previously mentioned processes may impede the construction of coherent narratives and therefore may be considered antecedents of low memory coherence.

Within the CaR-FA-X model, rumination, cognitive avoidance, and impaired executive functioning are described as contributors to reduced memory specificity without specifying the exact role of each of these constructs. Later research has built on this model by demonstrating that rumination in particular is more than merely a correlate of reduced memory specificity, but functions as a mediator of the relation between overgeneral memory and psychopathology [28,29]. In what will follow, we will describe how each of these three constructs may predict low memory coherence and we will review empirical evidence supporting this hypothesis.

### Rumination

According to the Response Styles Theory of Nolen-Hoeksema [30], rumination can be conceptualized as the continuous dwelling on one’s negative thoughts and feelings, their causes, and consequences. It is considered a vulnerability factor for the development and maintenance of major depressive disorder [31,32]. We hypothesize that this negative repetitive thinking style might impede the construction of coherent narratives in various ways. First, as described by Williams and colleagues [4], rumination prone individuals can get captured by general information (e.g., all the times I failed) that trigger rumination (e.g., “Why I am such a failure”, “I am a disappointment”) when engaged in the top-down, vertical search process for specific information in the ESK. While ruminating about this general and abstract information, the search for specific information will be truncated prematurely, consequently hindering the construction of a coherent narrative.

Second, the focus on the causes and consequences of negative affect instead of solutions, which is characteristic for rumination [30], will likely obstruct the creation of a resolution. Being able to formulate a resolution, which is a requirement for thematic coherence, requires sufficient emotional problem solving and a sophisticated set of metacognitive skills such as self-reflection and self-understanding [10]. The problem-focused instead of problem-solving nature of rumination might therefore impede the formulation of a resolution, resulting in lower coherence [33,34]. So, taken together, rumination may be considered an antecedent of low memory coherence.

Albeit limited, previous research seems to support the hypothesis that rumination relates to lower memory coherence. For example, a large-scale study in a sample of New-Zealand adolescents demonstrated that adolescents who indicated to ruminate less were able to construct more coherent narratives [15]. In addition, a prospective study by Vanderveren, Bijttebier, and Hermans [14] revealed rumination to be a mediator of the relation between low memory coherence and subjective distress following a negative life experience. This seems to suggest that rumination might be more than merely an antecedent of low memory coherence and might underlie the relation between the latter and psychopathology.

### Cognitive avoidance

As previously proposed, creating a coherent narrative about a personal experience implies accessing specific information concerning that experience. The individual must be willing to retrieve and thus be exposed to the details and emotional content of the experience asked to narrate upon. In the case of very negative or traumatic experiences, this process can elicit strong negative affect that can sometimes be overwhelming. Avoiding to be confronted with such negative affect might therefore impede the construction of a coherent narrative and might, in other words, be considered an antecedent of low memory coherence.

Whereas no empirical evidence regarding the link between cognitive avoidance and memory coherence exists to this date, research within the domain of memory specificity might provide some valuable insight. The affect-regulation hypothesis states that retrieving overgeneral memories can be considered an avoidant coping strategy that prevents the individual from being exposed to specific and potentially threatening information related to a personal experience [4,35]. In line with this hypothesis, individuals who indicate to engage more in avoidant coping strategies retrieve less specific autobiographical memories [36,37]. Given the overlap, both theoretically and empirically, between memory specificity and memory coherence [16,26], these findings provide some indirect support for our hypothesis.

### Executive functioning

The development of memory coherence is dependent on the development of various higher-order cognitive skills [10]. Impaired executive functioning could therefore affect the ability to construct coherent narratives in various ways or, in other words, could be considered an antecedent of low memory coherence. As we proposed earlier, a coherent narrative implies access to specific information regarding the event that is being narrated upon. As described by Williams and colleagues [4], when attempting to access specific information in the ESK, sufficient executive capacity is required to focus this top-down search on retrieving information related to the event in question, while simultaneously inhibiting irrelevant information. If for any reason one’s executive capacity is impaired, this could then result in less coherent narratives. However, more is needed in order to construct coherent narratives than solely access to specific information. Impaired executive functioning could likewise impede temporal ordering, perspective taking, or emotional problem solving, which are in addition to accessing specific information required in order to construct coherent narratives [10].

In correspondence with this hypothesis, Klein and Boals [38] showed that over the course of an expressive writing intervention, working memory capacity increases and narratives become more coherent. This gain in working memory capacity was significantly related to the increase in memory coherence. Moreover, a recent study by Vanderveren, Aerts, Rousseaux, Bijttebier, and Hermans [39] showed that students who performed better on a working memory task were able to create more coherent narratives.

### Present study

The aim of the present study was twofold. First, the relation between memory coherence and psychopathology, more specifically depression and PTSD, was examined. In line with previous studies, we predicted that being able to construct more coherent narratives, especially about negative experiences, would be related to less depressive and PTSD-related symptoms. In addition, individuals who met the clinical cut-off threshold for either depression or PTSD were predicted to construct less coherent narratives. Second, the associations between memory coherence on the one hand and rumination, cognitive avoidance, executive functioning, and meaning making on the other hand, were explored. The following predictions were formulated. We hypothesized a negative association between memory coherence and both rumination and cognitive avoidance. In addition, a positive relation between the former and both executive functioning and meaning making was predicted. With regards to rumination, we additionally predicted that this negative repetitive thinking style would mediate the association between memory coherence, more particularly coherence of negative narratives, and depressive symptoms.

## Method

### Participants

Data were collected from a community sample consisting of 355 Americans aged between 21 and 73 years old (*M* = 38.71, *SD* = 11.53), of whom 147 men (41.4%) and 208 women (58.6%). A total of 66 participants (18.6%) met clinical cut-off thresholds for major depressive disorder and 53 participants (14.9%) met clinical cut-off thresholds for PTSD.

### Procedure

Participants were recruited through Amazon’s online data collection platform Mechanical Turk (MTurk), which has been established to be a psychometrically reliable and valid recruitment tool [40,41]. Registered workers filled out an online survey, which was programmed in Qualtrics, after giving their informed consent. First, participants were asked to reminisce about two personal experiences. Afterwards, they completed a series of questionnaires. Participants were financially compensated after completion to the amount of $4. This study was approved by the Social and Societal Ethics Committee of KU Leuven and was preregistered on AsPredicted.org (http://aspredicted.org/blind.php?x=j739wh).

### Measures

#### Memory coherence

Participants were asked to write about one very negative and one very positive personal experience. The order in which these narratives were prompted (negative or positive memory first) was counterbalanced. These narratives were later coded by an independent rater using the multidimensional coding scheme developed by Reese and colleagues [10]. Scores for the three dimensions (i.e., context, chronology, and theme) were added up for both the positive and negative narratives. Additionally, an overall memory coherence score was calculated by adding up the former two. This coding scheme has been demonstrated to be a reliable measure for memory coherence in different samples and age groups [10,12]. Inter-rater reliability analysis on 10% of the narratives indicated good to substantial reliability, κ = .74 for context, κ = .79 for chronology, and κ = .86 for theme.

#### Depression

The presence of depressive symptoms was assessed by the depression scale of the *Depression Anxiety Stress Scale-21* (DASS-21) [42]. *The Patient Health Questionnaire-9* (PHQ-9) [43], which uses a clinical cut-off threshold of 10, was administered as well. Both the DASS-21 and the PHQ-9 have been shown to be reliable and valid instruments to measure depression [44,45].

#### PTSD

The *Impact of Event Scale* (IES) [46] was used to measure symptoms related to PTSD. In addition, the *Post-Traumatic Checklist-5* (PCL-5) [47] was administered, which measures PTSD symptoms and has a clinical cut-off threshold of 33 (any later reference to depression or PTSD diagnosis refers to individuals who met the clinical cut-off threshold for depression or PTSD). Participants were instructed to answer these questionnaires with the negative experience they described earlier in mind. Psychometric quality of both the IES and PCL-5 have been established by previous studies [48,49].

#### Rumination

The ten-item version of the *Ruminative Response Scale* (RRS) [50] was administered to assess rumination. Participants are asked to indicate how often they generally demonstrate a certain behavior in response to feelings of sadness or depression on a scale ranging from 1 (*almost never*) to 4 (*almost always*). More rumination is reflected by a higher score. The brooding scale was used for the analyses, for brooding is generally assumed to reflect the maladaptive component of rumination [51]. Previous studies have established the RRS to be a reliable and valid measure of rumination [50].

#### Cognitive avoidance

The *Cognitive Behavioral Avoidance Scale* (CBAS) [52] was used to assess cognitive avoidance. The CBAS consists of 31 items that measure both cognitive and behavioral avoidance, though the focus of the current study was on cognitive avoidance in particular. Participants rate the applicability of each item on a five-point scale, ranging from 1 (*not at all true for me*) to 5 (*extremely true for me*), with higher scores indicating more cognitive avoidance. The validity and reliability of the CBAS have been demonstrated by previous studies [52].

#### Executive functioning

Two distinct measures were administered to assess executive functioning, as it refers to a variety of higher-order cognitive skills. The *Effortful Control Scale* (ECS) [53] was used to assess self-regulation, which is one component of executive functioning. According to Rothbart and Posner [54], effortful control encompasses various self-regulatory skills such as sustained attention, inhibition, and behavioral activation. When filling out the ECS, participants are asked to indicate how well each statement describes themselves on a scale from 1 (*extremely untrue*) to 7 (*extremely true*). A higher score indicates more effortful control. In addition, participants completed a *Stroop Interference Test*, which measures selective attention, cognitive flexibility, and inhibition. Reaction time is measured during completion of the task, with faster reaction time being indicative of better executive functioning [55].

#### Meaning making

Meaning making was assessed by three questionnaires tapping into different aspects of meaning making; *Posttraumatic Growth Inventory* (PTGI) [56], *Benefit Finding Scale* (BFS) [57], and both the positive reappraisal and acceptance scale of the *COPE Inventory* [58]. For each of these measures, a higher score is indicative of more meaning making. Psychometric quality of these questionnaires has been demonstrated by previous studies [59,60].

### Data-analysis

SPSS 25.0 was used to analyze the collected data. We conducted the following preregistered analyses. Pearson correlation coefficients were calculated to examine the associations between memory coherence on the one hand and depression, PTSD, rumination, cognitive avoidance, executive functioning, and meaning making on the other hand. To examine differences between participants who met the clinical cut-off threshold for depression or PTSD and participants who did not in terms of their memory coherence, independent sample t-tests were performed. We performed mediation analyses using the PROCESS macro developed by Hayes [61]. We conducted two additional analyses that were not preregistered. We calculated partial correlation coefficients between memory coherence on the one hand and depression, PTSD, cognitive avoidance, executive functioning, and meaning making on the other hand, while controlling for rumination. Finally, we performed two binary logistic regression analyses with diagnostic status (depression and PTSD) as criterion and memory coherence and rumination as predictors.

## Preregistered analyses

### Results

#### Descriptive results

Internal consistency, mean, and standard deviation of all variables are presented in Table 1. Paired sample t-tests revealed that narratives describing a negative event were more coherent than narratives describing a positive event, *t*(354) = 5.52; *p* < .001, *d* = 0.29. On a sublevel, the former were more chronologically, *t*(354) = 2.56, *p* = .01, *d* = 0.14, and contextually coherent, *t*(354) = 6.68, *p* < .001, *d* = 0.36, though not more thematically coherent, *t*(354) = .45, *p* = .65, *d* = 0.02. Independent sample t-tests also showed an effect of gender, with men being less coherent than women (see Table 1). Additionally, there was an effect of age, with memory coherence increasing with age, *r*(353) = .16, *p* < .01.

**Table 1:** Descriptive information.

	*Descriptives*			*Gender differences*				
				*Men n = 147*		*Women n = 208*		
	*α*	*M*	*SD*	*M*	*SD*	*M*	*SD*	*t*(df)
MEMCO_NEG	-	1.71	.66	1.61	.64	1.77	.66	-2.31*(353)
MEMCO_POS	-	1.50	.61	1.41	.56	1.56	.63	-2.21*(353)
MEMCO_TOT	-	1.60	.52	1.51	.51	1.66	.53	-2.74**(353)
DASS	.95	.53	.74	.56	.76	.52	.73	.48(352)
PHQ	.92	5.05	5.80	4.87	5.88	5.17	5.74	-.48(352)
IES	.95	.76	.79	.76	.82	.76	.78	.04(351)
PCL	.96	12.88	16.04	12.78	15.93	12.95	16.15	-.10(351)
Rumination	.86	.92	.76	.83	.75	.98	.77	-1.91(351)
Cognitive Avoidance	.95	.76	.81	.82	.85	.73	.78	1.02(350)
Effortful Control	.84	5.01	.96	5.04	.99	4.98	.94	.54(304)
Stroop RT^a^	-	23.53	11.85	23.28	12.75	23.70	11.20	-.31(316)
Benefit Finding	.96	1.94	1.12	1.82	1.02	2.03	1.19	-1.82^b^(336.17)
Acceptance	.89	2.13	.74	2.11	.71	2.14	.76	-.37(348)
Positive Reappraisal	.85	1.77	.84	1.70	.79	1.82	.88	-1.33(348)
Posttraumatic Growth	.96	2.35	1.38	2.19	1.30	2.47	1.43	-1.89(348)

MEMCO_NEG = coherence of negative narratives; MEMCO_POS = coherence of positive narratives; MEMCO_TOT = total memory coherence; DASS = Depression Anxiety Stress Scale; PHQ = Patient Health Questionnaire; IES = Impact of Event Scale; PCL = Post-Traumatic Checklist; Stroop RT = reaction time on Stroop task.

^a^ Reaction time in seconds.

^b^ Independent t-test adjusted for unequal variances across gender.

* *p* < .05

** *p* < .01.

#### Memory coherence, depression, and PTSD

Pearson correlation coefficients showed no significant associations between memory coherence and depressive or PTSD-related symptoms (see Table 2). In addition, independent sample t-tests revealed that participants who met the clinical cut-off threshold for major depressive disorder (*M* = 1.57, *SD* = .54) were not less coherent compared to participants who did not (*M* = 1.61, *SD* = .52), *t*(352) = .60, *p* = .55, *d* = 0.08. Similarly, participants who met the clinical cut-off threshold for PTSD (*M* = 1.51, *SD* = .52) were not less coherent than participants who did not (*M* = 1.62, *SD* = .52), *t*(351) = 1.40, *p* = .16, *d* = 0.21.

**Table 2:** Overview Pearson correlation coefficients.

	MEMCO_NEG	MEMCO_POS	MEMCO_TOT
DASS	-.04	-.07	.-07
PHQ	.04	-.06	-.01
IES	.04	.01	.03
PCL	-.02	-.06	-.05
Rumination	.12*	-.02	.07
Cognitive Avoidance	-.06	-.07	-.08
Effortful Control	.07	.16**	.14*
Stroop RT	.04	.08	.07
Benefit Finding	.15**	.15**	.18**
Acceptance	.17**	.17**	.20***
Positive Reappraisal	.07	.12*	.11*
Posttraumatic Growth	.10	.14**	.14**

MEMCO_NEG = coherence of negative narratives; MEMCO_POS = coherence of positive narratives; MEMCO_TOT = total memory coherence; DASS = Depression Anxiety Stress Scale; PHQ = Patient Health Questionnaire; IES = Impact of Event Scale; PCL = Post-Traumatic Checklist; Stroop RT = reaction time on Stroop task.

* *p* < .05

** *p* < .01

*** *p* < .001

#### Memory coherence, rumination, cognitive avoidance, executive functioning, and meaning making

The coherence of narratives describing a negative experience was significantly associated with rumination, with higher coherence being related to more rumination. There was no significant association found between memory coherence and cognitive avoidance. With regards to executive functioning, higher total memory coherence and coherence of memories describing a positive experience were significantly related to more effortful control, yet no significant association with performance on the Stroop task was observed. Finally, concerning the relation with meaning making, more memory coherence was related to more benefit finding, acceptance, positive reappraisal, and post-traumatic growth. Coherence of positive narratives showed similar associations with meaning making, though coherence of negative narratives was exclusively associated with benefit finding and acceptance (cfr. Table 2).

#### Rumination as potential mediator

As there was no significant association between coherence of negative narratives and depressive symptoms, we could not investigate rumination as a potential mediator of this association. However, we could investigate a possible indirect effect between memory coherence and depression through rumination. According to Holmbeck [62], two types of intervening effects exist, namely a direct and an indirect effect. In the case of a direct effect, also referred to as a mediated effect, a direct association between the independent and the dependent variable exists. This direct association is partially or fully explained by a third variable, the mediator. When the mediator is added to the model and the shared variance between the independent variable and the mediator is accounted for, the association between the former and the dependent variable partially or completely disappears. In the context of an indirect effect, the association between the independent and dependent variable only exists through an intervening variable [61]. When the shared variance between this variable and the independent variable is adjusted for, a significant association between the independent and the dependent variable appears [63].

An examination of this potential indirect effect indicated that, when the effect of rumination was adjusted for, higher coherence of negative narratives was significantly associated with less depressive symptoms. So, there was a significant indirect effect between coherence of negative narratives and depressive symptoms through rumination. When repeating this analysis for PTSD-related symptoms, we likewise found that higher coherence of negative narratives significantly related to less PTSD-related symptoms when controlling for rumination.

These surprising findings can be interpreted as follows. The negative relation between memory coherence and depressive symptoms only becomes apparent when rumination is taken into account. When the shared variance between memory coherence and rumination is accounted for, an indirect effect between the former and depressive symptoms appears. This negative relation could previously not be observed due to the shared variance or overlap between memory coherence and rumination. This positive relation with rumination, which on its own is positively related to depressive symptoms, cancelled out the negative association between memory coherence and depressive symptoms. The piece of memory coherence we are then left with, which cannot be explained by rumination, is negative related to depressive symptoms. The same interpretation holds for PTSD-related symptoms.

## Exploratory analyses

The previously reported results indicated that, contrary to predictions, the ability to construct coherent narratives is not related to depression or PTSD. In addition, the predicted negative association between memory coherence and both rumination and cognitive avoidance could likewise not be confirmed. Moreover, results indicated that more memory coherence is related to *more* rumination. Surprisingly, a significant indirect effect between memory coherence and both depressive and PTSD-related symptoms through rumination was observed. In light of these unexpected findings, the analyses were repeated on an exploratory basis, but now with rumination taken into account.

### Results

#### Memory coherence, depression, and PTSD

Table 3 contains an overview of partial correlation coefficients with rumination as a control variable. When controlling for rumination, higher memory coherence was related to less depressive symptoms and symptoms of PTSD. Coherence of negative narratives showed the same association with depressive symptoms and PTSD symptoms, though the coherence of positive narratives was not significantly associated with either of these symptoms. In addition, two binary logistic regression analyses were performed to assess whether PTSD and depression diagnosis could be predicted by the coherence of negative narratives when controlling for rumination. Regarding PTSD diagnosis, results of the binary logistic regression indicated that coherence of negative narratives (Wald χ^2^ = 4.93, *p* = .03, OR = .54, 95% CI: .31 to .93) could predict PTSD diagnosis after controlling for rumination (Wald χ^2^ = 59.83, *p* < .001, OR = 7.78, 95% CI: 4.63 to 13.09). When repeating this analysis for depression diagnosis, results demonstrated that coherence of negative narratives (Wald χ^2^ = 1.84, *p* = .18, OR = .73, 95% CI: .47 to 1.15) could not significantly predict depression diagnosis after controlling for rumination (Wald χ^2^ = 45.45, *p* < .001, OR = 3.79, 95% CI: 2.57 to 5.57).

**Table 3:** Overview of partial correlation coefficients after controlling for rumination.

	MEMCO_NEG	MEMCO_POS	MEMCO_TOT
DASS	-.16**	-.11	-.16**
PHQ	-.03	-.07	-.06
IES	-.07	.01	-.04
PCL	-.15**	-.07	-.14*
Cognitive Avoidance	-.16**	-.10	-.16**
Effortful Control	.13*	.17**	.18**
Stroop RT	.05	.08	.08
Benefit Finding	.13*	.13*	.16**
Acceptance	.18**	.15*	.20**
Positive Reappraisal	.07	.11	.11
Posttraumatic Growth	.07	.12*	.11

MEMCO_NEG = coherence of negative narratives; MEMCO_POS = coherence of positive narratives; MEMCO_TOT = total memory coherence; DASS = Depression Anxiety Stress Scale; PHQ = Patient Health Questionnaire; IES = Impact of Event Scale; PCL = Post-Traumatic Checklist; Stroop RT = reaction time on Stroop task.

* *p* < .05

** *p* < .01

#### Memory coherence, cognitive avoidance, executive functioning, and meaning making

When controlling for rumination, total memory coherence and coherence of negative narratives was negatively associated with cognitive avoidance. With regards to executive capacities, higher memory coherence, coherence of positive and negative narratives was related to more effortful control, though no significant association with performance on the Stroop task was observed. Finally, memory coherence was positively related to two distinct components of meaning making after controlling for rumination; benefit finding and acceptance. Coherence of negative and positive narratives showed similar positive associations with meaning making (cfr. Table 3).

## Discussion

The purpose of the present study was twofold. First, the relations between memory coherence and both depression and PTSD were examined. Second, the associations between memory coherence on the one hand and rumination, cognitive avoidance, executive functioning, and meaning making on the other hand, were investigated. In addition, rumination was examined as a potential mediator of the relation between depression and memory coherence.

With regards to the first research question, the ability to construct coherent narratives was, contrary to predictions, unrelated to the presence of depressive or PTSD-related symptoms. Furthermore, individuals who met the clinical cut-off threshold for depression or PTSD were not less coherent compared to individuals who did not. Concerning the role of rumination, cognitive avoidance, executive functioning, and meaning making, results were as follows. The hypothesized negative relation between memory coherence and both rumination and cognitive avoidance could not be confirmed. Moreover, results indicated that more memory coherence actually related to *more* rumination. The observed positive association between memory coherence and both executive functioning and meaning making were in correspondence with predictions. More memory coherence was related to more effortful control, though no significant association with performance on the Stroop task was observed. With regards to meaning making, the ability to construct coherent narratives was related to more acceptance, positive reappraisal, benefit finding, and post-traumatic growth. Finally, the indirect effect between memory coherence and both depressive and PTSD-related symptoms through rumination was examined. Results indicated that memory coherence was in fact negatively related to depression and PTSD when rumination was taken into account. Interpretation of these rather surprising findings will be provided later on in the discussion.

In light of the surprising indirect effect between memory coherence and symptoms of depression and PTSD through rumination, some additional exploratory analyses were conducted. When controlling for rumination, memory coherence was predictive of PTSD diagnosis, though not of depression diagnosis. Moreover, more memory coherence was related to less cognitive avoidance after controlling for rumination. Relations between memory coherence on the one hand and executive functioning and meaning making on the other hand remained similar after controlling for rumination. As these analyses were exploratory and post-hoc, caution in interpreting these results as well as replication of these findings are required.

Perhaps the most intriguing observation is that a ruminative thinking style actually relates to *more* memory coherence. In fact, we hypothesized that the abstract and problem-focused nature of rumination would impede the construction of coherent narratives by hindering access to specific information and by impeding the forming of a resolution. In order to grasp this surprising observation, we should first capture what rumination precisely entails. High ruminating individuals have the tendency to repeatedly dwell on the causes and consequences of the experiences they encounter. They analyze and reanalyze the circumstances surrounding an experience with an emphasis on their negative affect [30]. Even though this manner of repeated thinking is neither productive nor solution-oriented, it does result in a highly rehearsed and consolidated account of the events, rich in emotional elaboration. When asked to narrate about a significant personal experience, high ruminating individuals will then likely be able to construct a coherent narrative due to the extensive mental exercise. However, it is most likely not their general ability to construct coherent narratives that is being measured, but rather the product of repeated rumination. Results of the present study seem to suggest that when this degree of mental exercise is not taken into account, and therefore memory coherence is not being measured as a true cognitive skill, its true association with psychopathology is obscured. In contrast, when rumination is controlled for, the cognitive ability to construct coherent narratives does appear to be significantly related to both depression and PTSD.

The present study sheds an interesting light on the construct of memory coherence and its relation to psychological well-being and psychopathology. Developmental research has demonstrated that memory coherence can be considered an individual difference factor, with some individuals generally being more capable of constructing coherent memories than others [10,64,65]. In line with this notion of memory coherence as a cognitive skill, is the observation that individuals’ memory coherence is consistent over time and across memories [64,65]. In addition to being a cognitive skill, research has indicated that memory coherence is dependent on specific characteristics of the event being narrated upon, such as valence, intensity, and time passed since occurrence of the event. For example, memories about negative experiences are generally more coherent than memories about positive experiences [16,66]. So, it appears that memory coherence is an individual difference factor, though dependent on event-specific characteristics as well [65]. Yet, research aimed at investigating the relation between memory coherence and psychological well-being typically focusses on how the *coherence of a particular memory* is associated with *well-being regarding that specific experience*. We propose, however, that someone’s *ability to construct coherent memories* relates to his or hers *well-being in general*, irrespective of the specific memory being recalled. Results from a recent prospective study seem to support this alternate approach by showing that being able to construct coherent narratives protects the individual from the potential harmful impact of *future* stressors, suggesting memory coherence makes individuals more resilient in general [14]. It would be a valuable addition to the literature to approach memory coherence as a cognitive skill that relates to well-being and psychopathology in general, rather than solely focusing on how the coherence of a particular memory is instrumental in dealing with that specific event.

It is important to note that the results of the present study should be interpreted with the necessary caution given the exploratory nature of the analyses. Nevertheless, the results appear promising from a clinical point of view. The current study contributes to the growing belief that the ability to construct coherent narratives about significant personal experiences is beneficial for people’s psychological well-being by demonstrating that individuals who don’t master this skill, report more depressive and PTSD-related symptoms. If additional research would support this claim that low memory coherence poses a vulnerability for developing psychopathological symptoms, a clinically valuable extension to this research line could be to explore the efficacy of a memory coherence training as a primary prevention or add-on intervention.

The focus on correlates of memory coherence and its relation with psychopathology, as well as its large sample size, are definite strengths of the present study. Nonetheless, some limitations should be acknowledged. First, no data about when the narrated events occurred, were collected. The age of the memory could be an important confounding variable, as previous research has indicated that memory coherence can change over time [23,38]. More opportunity for emotional processing due to more time between occurrence and recall of the event, might lead to more coherence. This could have an influence on the relation between coherence and well-being. We therefore recommend future studies in this area to take the age of the memory into account.

Another noteworthy limitation to the present study is its cross-sectional design, which does not allow us to make statements regarding the direction of the relation between memory coherence and both depression and PTSD. It is possible that the presence of depressive or PTSD-related symptoms are a consequence of low memory coherence, though the latter could likewise be a product of these symptoms. Empirical evidence appears to lend support for both hypotheses, suggesting the relation is bidirectional. In the prospective study by Vanderveren, Bijttebier, and Hermans [14] we cited to earlier, results seemed to suggest that students low in memory coherence were more vulnerable to the psychological impact of negative life events. Yet an experimental mood induction study demonstrated that inducing a negative mood (one of the core symptoms of depression) in women subsequently affects their ability to construct coherent narratives [39]. A large-scale longitudinal study in which the temporal relations between memory coherence and symptoms of psychopathology are investigated, could provide valuable insight into this matter. Similarly, such a design would allow us to further investigate the exact role of rumination, cognitive avoidance, executive functioning, and meaning making.

However, given the results of the present study, it would be recommended to adjust the design of future studies in such a way that memory coherence is being assessed as a cognitive skill rather than the product of repeated thinking, as the latter could obscure some important associations. To obtain this, a design in which participants are prevented from ruminating about the experience they will be asked to narrate upon, is required. One possibility could be to experimentally induce a significant personal experience (e.g., stressful task, failure, social rejection) that participants are then immediately instructed to narrate about. That way, participants won’t have the opportunity to ruminate about the experience before being asked to construct a narrative about it. Coherence of this writing assignment could then be investigated in relation to the psychological impact and processing of the experience. So, when conducting fundamental research regarding memory coherence in relation to psychopathology and potential underlying mechanisms of this relation, measuring memory coherence as a cognitive skill could be critical.
